# Intelligence, Correspondence, &c.

**Published:** 1839

**Authors:** 


### INTELLIGENCE, CORRESPONDENCE, &c.

The ironical praise of Animal Magnetism, in our Irish friend's letter, is at least ten months too late. The humbug is blown to the winds, and the hole-and-corner Saturnalia, which are now carried on, will soon follow.

We shall be happy to hear again from the "Market-hill Dispensary," when any interesting matters turn up.

New York Quarterly Journal of Medicine and Surgery.

☞ The First Number of this new cotemporary is to appear on this day, 1st July, 1839, under the auspices of numerous and able Collaborateurs. We accept with pleasure the offer of mutual exchange, and shall be happy to introduce our young friend on all occasions. We see Mr. Churchill, of London, is the Agent in this our Old World.

